# Madras Medical Reports

**Published:** 1832-10-01

**Authors:** 


### Medical Reports, selected by the Madras Medical Board, from the Records of their Office, &c. Madras, 1831.

These reports are drawn up by Mr. Fleming, the Secretary of the Board, and are necessarily miscellaneous in their nature and unequal in their importance. The first four reports from Messrs. Lister, Thomson, Lawrance, and Scarman, are on epidemic cholera; but unfortunately we have enough of that article now at home, without any necessity for a fresh *importation.* It is not a little curious, however, to remark that the epidemic cholera which has occurred of late years in India, corresponds much more with that which is ravaging Europe, than that which has been described by the three Boards soon after 1817. This similarity respects the antecedent diarrhœa and the consecutive fever, which now seem to be almost as constant in India as in Europe. This fact alone goes far to shew that epidemic causes are at work on a large scale, and one larger than formerly. The following extract from one of these reports is worthy of record.

"Causes. The regiment left Bellary on the 4th October; and, from that day until the 26th, when it crossed the Kistna, the rain was almost incessant, with much thunder. We were detained seven days on the banks of the Hoogery, two at the Toomboodera, and three at the Kistna. The men suffered little sickness during this time; but I imagine the fatigue and exposure they then underwent predisposed them to the disease. Many of them were greatly in want of money, and, I fear, were consequently very badly fed. After crossing the Kistna we had no more rain; the sun was powerful during the day, and the nights were very cold. Many of the patients said they had slept exposed to the open air at night. This is all I observed respecting the cause of this severe attack. There was no cholera in any of the villages we passed. A great many of the followers were attacked, and suffered more severely than the sepoys, not being brought to the hospital till past recovery. 25 cases among the sepoys occurred after our arrival here, and two as late as 13 days afterwards. There was not a case of cholera except in our lines at this station. These facts show the length of time the disposition to the disease may continue in those who have been exposed to its causes. A very remarkable instance of the same kind was mentioned to me by Mr. M'Cabe, which it may not be improper to insert here. A number of Europeans came from St. Thomas's Mount, where the cholera was raging, to Poonamallee, which was free from the disease. In the course of a few days 22 of them were seized with cholera, whilst *not one* of the other Europeans at Poonamallee were attacked. These facts seem also to argue strongly that the disease is not contagious. With respect to sol-lunar influence, these cases favor Mr. Orton's opinion, as 39 of them occurred near the full and change of the moon, and only 14 near the quarters." 16.

A remarkable instance of the effects of locality is mentioned by Mr. Lister of the 46th Regiment. In the month of August, 1826, a detachment, arrived from Bellary on the right bank of the Kistna. The river had overflowed its banks, and the men were delayed for want of boats on the oozy soil for a day or two. The cholera immediately broke out; and continued till they crossed the river and got to some distance on the other side, when it disappeared. Two officers who were very much afraid of the disease caught it, and one of them died. Many other instances are related by the different reporters of the injurious influence of rivers and malarious localities, as also of the strong predisposition caused by fear. We could add many similar instances in this metropolis, both of the tenacity with which the disease clings to the banks of Rivers, and the sedative effects of terror as predisposing to the choleric orgasm. As to the remedies employed by the Madras Reporters, we need not enumerate them here. Few of them were useful—none specific.

II. *Dysentery*. Dr. Mortimer, in a report, draws the attention of the profession to the employment of ipecacuan in dysentery, given in nauseating doses immediately after bleeding, either general or topical, followed by a dose of castor oil. This remedy was exhibited to the extent of five grains (mixed with some powdered gum arabic) every hour or second hour, as the patient's stomach would bear it without actual vomiting. During the taking of this medicine, no fluids were permitted to be swallowed. The nausea is rather distressing, and vomiting is often induced though not desired. Sweat is generally excited. The improvement of the biliary secretion under the administration of ipecacuan was manifest. Where the bile was vitiated or redundant calomel, in considerable doses, was employed. This Report is drawn out to an unreasonable length by the unnecessary multiplication of cases, and the tediously minute detail of all the symptoms and remedies. Still the Report contains valuable materials for the Indian practitioner's guidance at the bed side.

III. *On the Use of Sulphate of Quinine.* By Surgeon W. Geddies *and others.*—Our author, who has used the quinine very extensively in India, affirms that, next to opium, it is the most valuable medicine we possess. Its efficacy in checking the tendency to paroxymal exacerbations of disease, so general in tropical climates, renders it an invaluable remedy there as well as elsewhere.

"The types of constitutional affection, attendant on dysentery, irritation, debility, and the like, all appear, at times, to put on this paroxysmal form; and, as tending much to aggravate the original disorder, it is evidently an important point, in the treatment of them, to check the febrile exacerbations at as early a period as possible. Wherever, therefore, in any disease, an evident increase of the frequency of pulse, and heat of skin, has been observed to occur, at any particular period of the day, for two or more times, I have at once, without reference to the presence of any other symptoms, had recourse to the quinine, in combination with other remedies for the relief of whatever other disorder may attend the febrile exacerbations. I have thus used the quinine, not only in intermittent fever, or, its more violent form, the remittent type, but I have extended its use to check the exacerbations of the febrile affection attending, and so greatly aggravating, acute dysenteric affections. I have also applied it to prevent the quotidian exacerbations, occasionally occurring in convalescence from tedious, or severe, disorders, such as hepatitis, chronic degrees of dysentery or diarrhœa, bad health with ulcers, and the like. I have used it likewise for the relief of some other disorders of an intermittent nature, and for rheumatism; and its tonic powers have been applied for various states of the constitution requiring such remedies." 202.

Mr. Geddes proceeds to the particulars of his treatment; but they need not detain us here, as the principle is easily understood, and the application is now in common use among the best informed practitioners.

This report is followed by one from assistant Surgeon Malcolmson, on the same subject, and proving the great value of the quinine, in tropical fevers of intermittent type. Mr. Cruikshank succeeds Mr. Malcolmson, and these two are followed by several other reporters, all bearing the same testimony.

IV. *Cases of Phlebitis.* By SURGEON BOND. Several cases of this dangerous affection are related, one or two of which are fatal—the rest successful, apparently from an operation which Mr. Bond got into the habit of performing in such cases. The operation is thus concisely described by one of the members of the Madras Medical Board, who was superintendent surgeon of the station, where Mr. Bond practised, and witnessed the cases.

"The operation was performed as follows. An incision was made with a scalpel through the integuments, and the median basilic vein exposed, a director was then introduced through the puncture, and, with a probe pointed bistoury, the opening was extended above and below as far as there was any appearance of pus, which was carefully extracted by pressure, and the wound closed in the usual manner." 271.

V. *Syphilis treated without Mercury.* A long train of reports are here presented on a subject that excited considerable interest a few years ago. We believe that few practical surgeons, now-a-days, trust to the non-mercurial treatment of syphilis, where the disease is unequivocal. Nevertheless, the experiments and investigations on this subject, have greatly checked the abuse of mercury in syphilitic complaints, though they have not shaken the faith of surgeons in a well regulated administration of this powerful medicine.

The volume concludes with some additional reports on the use of quinine, and a short paper by Mr. Lane, on purulent and gonorrhœal ophthalmia. We beg to return our best thanks to the Madras Medical Board for the volume which we have thus hastily noticed.

